# Influence of Pulse Current Forward-Reverse Duty Cycle on Structure and Performance of Electroplated W–Cu Composite Coatings

**DOI:** 10.3390/ma14051233

**Published:** 2021-03-05

**Authors:** Yuchao Zhao, Nan Ye, Haiou Zhuo, Chaolong Wei, Weiwei Zhou, Jie Mao, Jiancheng Tang

**Affiliations:** School of Materials Science and Engineering, Nanchang University, No. 999, Xuefu Avenue, Nanchang 330031, China; 355729114008@email.ncu.edu.cn (Y.Z.); yenan8708@outlook.com (N.Y.); haiou_zhuo@ncu.edu.cn (H.Z.); 411314018079@email.ncu.edu.cn (C.W.); 411314018100@email.ncu.edu.cn (W.Z.); 411314019051@email.ncu.edu.cn (J.M.)

**Keywords:** W–Cu composite, electroplating, forward-reverse pulse current, duty cycle, microstructural uniformity, mechanical properties

## Abstract

Tungsten-copper (W–Cu) composites are widely used as electrical contact materials, resistance welding, electrical discharge machining (EDM), and plasma electrode materials due to their excellent arc erosion resistance, fusion welding resistance, high strength, and superior hardness. However, the traditional preparation methods pay little attention to the compactness and microstructural uniformity of W–Cu composites. Herein, W–Cu composite coatings are prepared by pulse electroplating using nano-W powder as raw material and the influence of forward-reverse duty cycle of pulse current on the structure and mechanical properties is systematically investigated. Moreover, the densification mechanism of the W–Cu composite coating is analyzed from the viewpoints of forward-pulse plating and reverse-pulse plating. At the current density (*J*) of 2 A/dm^2^, frequency (*f*) of 1500 Hz, forward duty cycle (*d_f_*) of 40% and reverse duty cycle (*d_r_*) of 10%, the W–Cu composite coating rendered a uniform microstructure and compact structure, resulting in a hardness of 127 HV and electrical conductivity of 53.7 MS/m.

## 1. Introduction

Tungsten-copper (W–Cu) composite is a pseudo-alloy, which is highly promising for a wide array of applications, such as electrical contact materials, resistance welding, electrical discharge machining (EDM), plasma electrode materials, electrothermal alloys, etc. Due to its high melting point and hardness of W and high electrical and thermal conductivity of Cu, W–Cu composites render excellent for erosion resistance, fusion welding resistance, high strength, and high hardness [1,2,3].

However, W–Cu composites prepared by liquid-phase sintering or infiltration exhibit inferior compositional uniformity and an undesirable contradiction between density and grain size due to the significant difference in melting points of W and Cu [4,5]. It has been reported that the reduction in the size of W particles or the addition of a small amount of nano-W powder can improve the hardness and conductivity of W–Cu composites [6,7,8]. However, with the decrease of W grain size, it is difficult to balance densification and microstructural uniformity. This results in inferior electrical and thermal conductivities. In addition, the stringent requirements of the sintering process during the conventional preparation methods limit the large-scale applications. Therefore, electroplating is introduced to prepare Cu-coated W powder, which is mixed sintering to obtain high-performance W–Cu alloys. However, the direct current (DC) electroplating can only result in a mixture of Cu and W, and the W–Cu composites cannot be directly prepared [1,9,10,11].

The utilization of pulsed current to electrodeposit metals and alloys can better control the properties of electrodeposited layers. Furthermore, the performance of resulting metals and alloys can be improved by altering the microstructure, which is achieved by adjusting the pulse parameters [12,13,14]. For instance, Thiemig et al. [15] have conducted extensive research on pulse electroplating of metals and alloys and demonstrated that the selection of pulse parameters renders a great influence on the composition of alloy coating. It has been reported that the employment of pulse current can significantly reduce the internal stress of electric casting compared with the conventional DC at the same average current density. Recently, Li et al. [16] have studied the effect of pulse electroplating on the surface roughness of electrodeposited nickel films. Marro et al. [17] also found the influence of pulse plating process parameters on the morphology and stress state of Cu film. The results showed when compared with DC electroplating, the electrodeposition of Cu by pulse electroplating could lead to minimal porosity and superior strength. However, there are few reports on the preparation of W–Cu composites by pulse plating, and the effects of pulse process parameters (pulse frequency, current density, duty cycle) on the structure and properties of W–Cu composites have not been studied [12,18,19]. Herein, we aim to directly prepare W–Cu composites by a one-step electroplating method using nano-W powder.

In previous research, using the single positive pulse power supply to adjust the pulse process parameters could not obtain a compact composite coating. By using double positive and negative pulse power supplies, increasing the reverse current plating, and adjusting the duty cycle of the positive and negative currents, a dense composite coating was obtained. It shows that the reverse current and the duty cycle of the forward-reverse current play an important role in the structure of the composite coating. Therefore, the influence of the duty cycle of the forward-reverse currents on the microstructure and properties of the W–Cu composite coating is discussed in detail in this article. Moreover, the densification mechanism is also elaborated from the viewpoints of forward-pulse plating and reverse-pulse plating.

## 2. Materials and Methods

**Figure 1:**
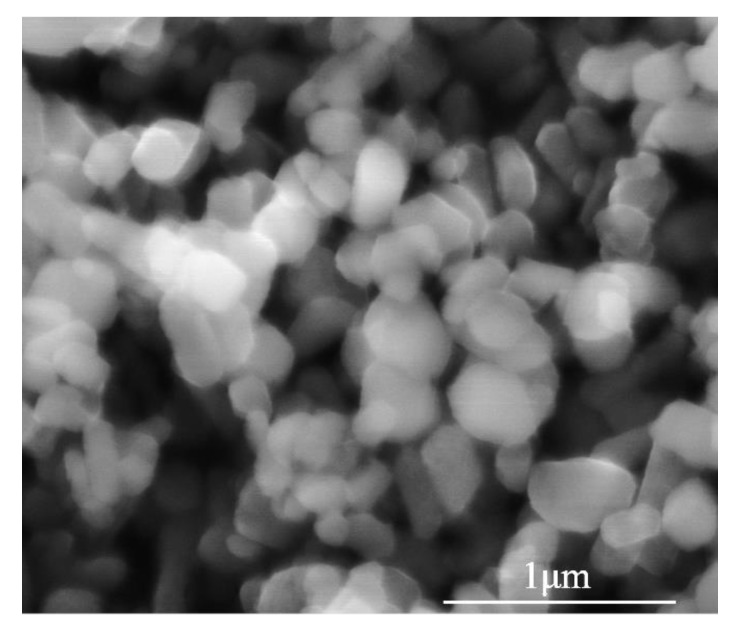
Scanning electron microscope (SEM) image of the nano-W powder.

In this study, the self-made W powder was used as a raw material prepared by carbon-assisted hydrogen reduction method [20]. The purity of W powder is 99.98%. The morphology (SEM, FEI, Hillsboro, OR, USA) of nano-W powder is shown in Figure 1, consisting of regular spherical particles with uniform size distribution and an average particle size of 200 nm.

**Figure 2:**
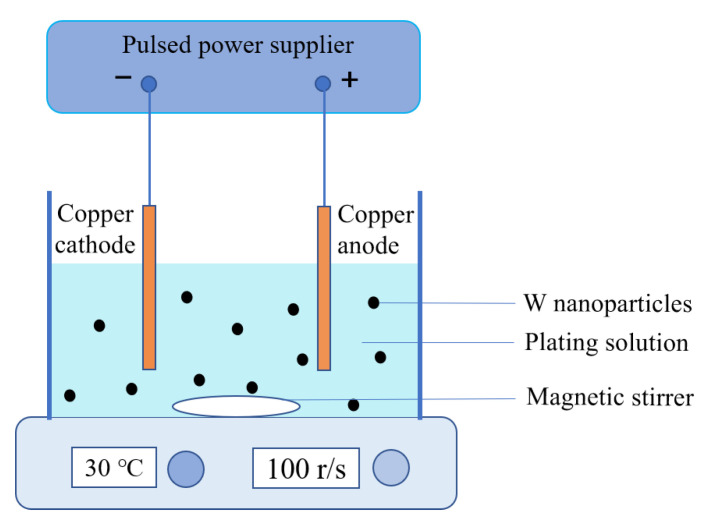
The schematic illustration of the pulse plating device.

**Figure 3:**
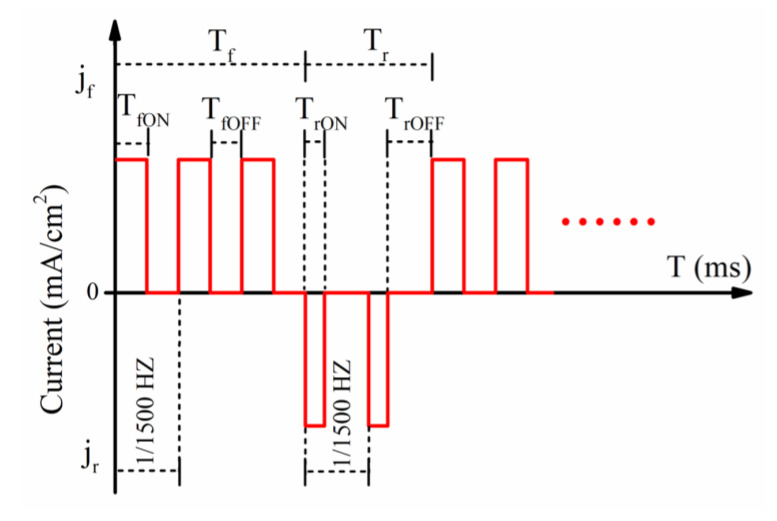
The schematic diagram of forward- and reverse-pulse currents to prepare tungsten-copper (W–Cu) composite coatings.

The experimental device used to prepare W–Cu composites is shown in Figure 2. The pulse rectifier (Yueyang, Ningbo, Zhejiang Province, China, Electric appliance, 60V/20A) was used as a pulse power supply, and Cu plates (~99.99%, dimension 40 mm × 30 mm × 0.5 mm, Jiangxi Copper Industry Group Co., Ltd., Nanchang, Jiangxi province, China) were used as the cathode and anode. A temperature-controlled magnetic stirrer (Hangzhou Ruijia Precision Scientific Instrument Co., Ltd, Hangzhou, Zhejiang Province, China) was used to maintain the temperature of the plating solution and to enhance the dispersion of W particles by stirring the plating solution. During pulse electroplating, when the current is turned from on-to-off in the forward direction, the pulse duration is called the forward pulse width (*T_fON_*). During this time, the possible reactions and standard electrode potentials *φ^Θ^* are as follows [21]: Cu^2+^ + 2e^−^ = Cu, *φ^Θ^* = 0.340 V; Cu^2+^ + e^−^ = Cu^+^, *φ^Θ^* = 0.158 V; Cu^+^ + e^−^ = Cu, *φ^Θ^* = 0.522 V; 2H^+^ + 2e^−^ = H_2_, *φ^Θ^* = 0.000 V. The larger the electrode potential is, the easier the electrons are to be reduced. Therefore, Cu^2+^ ions are reduced to Cu on the cathode Cu plate. The “off” duration is denoted as *T_fOFF_* and the ratio of *T_fON_*/(*T_fON_* + *T_fOFF_*) is called the forward duty cycle, which is denoted as *d_f_*. When the current is reversed, the “on” duration is called reverse pulse width, which is recorded as *T_rON_*. During this time, the Cu cathode is anodized and the Cu becomes Cu^2+^ ions, which are dissolved in the electroplating solution. The “off” duration is denoted as *T_rOFF_* and the ratio of *T_rON_*/(*T_rON_* + *T_rOFF_*) is called the reverse duty cycle (*d_r_*). The ON-time of the forward and reverse current is collectively called *T_ON_*, and the OFF-time is collectively called *T_OFF_*. Herein, the modulated pulse current was used for electroplating (Figure 3). According to the requirements of the pulse power supply, the forward current density is denoted as *j_f_* and the reverse current density is denoted as *j_r_*. They are collectively referred to as current density (*J*) and the pulse current density (*J*) was the average current density. The relationship between peak current density (*J_P_*) and average current density (*J*) and duty cycle (*d*) can be given as follows [22,23]:

(1) $$Jp\;=\;J/d\;=\;J\left(T_{ON}+T_{OFF}\right)/T_{ON}$$

The composition of the plating solution was CuSO_4_·5H_2_O (125 g/L), H_2_SO_4_ (200 g/L), NaCl (60 mg/L), polyethylene glycol (PEG, 0.2 g/L), and sodium dodecyl sulfate (SDS, 0.1 g/L). All chemical reagents are analytical reagents, provided by Sinopharm Chemical Reagent Company (Shanghai, China). Then, nano-W powder (10 g/L) was added to the plating solution for composite electroplating. The electroplating process parameters are shown in Table 1. The samples under different conditions were prepared by changing the forward and reverse duty cycle of the pulse current. Before electroplating, Cu plates were polished with a series of silicon carbide sandpaper (STARCKE MATADOR, Melle, Germany) in the order of 800, 1000, 1200, 1500 and 2000 mesh to prevent contaminations. Subsequently, H_2_SO_4_ (5 vol.%, Sinopharm Chemical Reagent Company, Shanghai, China) and HCl (10 vol.%, Sinopharm Chemical Reagent Company, Shanghai, China) solutions were used for activation. Finally, the Cu plate was cleaned using anhydrous ethanol (Sinopharm Chemical Reagent Company, Shanghai, China). After pulse plating, the samples were gently washed with absolute ethanol (Sinopharm Chemical Reagent Company, Shanghai, China) and dried in an oven at 60 °C.

Scanning electron microscope (SEM, FEI-quanta200f, Hillsboro, OR, USA) was used to observe the surface and cross-sectional morphologies. X-ray diffraction (XRD, brukerd8 X-ray diffractometer focusing (Billerica, MA, USA, Cu Kα radiations, 40 kV and 40 mA) was used for structural analysis. The high-resolution transmission electron microscopy (HRTEM, FEI Talos f200x, Hillsboro, OR, USA) was used to observe the electroplating layer. The hardness was measured with a microhardness tester (HVS-1000, Shanghai Precision Instrument Co., Ltd., Shanghai, China). The electrical conductivity was measured using a Sigma 2008a digital conductivity meter (Xiamen, Fujian province, China). ThermoICAP-6300 inductively coupled plasma spectrometer (ICP, Boston, MA, USA) was used for compositional analysis.

## 3. Results and Discussion

### 3.1. Effect of Forward Duty Cycle on the Structure of W–Cu Composite Coatings

**Figure 4:**
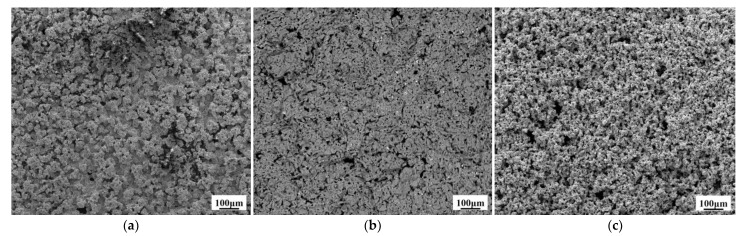
Surface SEM images of the W–Cu composite coatings under different forward duty cycles: (**a**) *d_f_* = 20%, (**b**) *d_f_* = 40%, and (**c**) *d_f_* = 60%, (*J* = 2 A/dm^2^, *f* = 1500 Hz and *d_r_* = 5%).

Figure 4 presents the effect of pulse current forward duty cycle on the surface morphology of W–Cu composite coatings. At *d_f_* = 20%, the composite surface was rough with obvious large granular protrusion. However, when the *d_f_* was increased to 40%, the composite surface was found to be much flatter without obvious granular structure. As the *d_f_* is further increased to 60%, the composite surface became loose with a high concentration of holes. As shown in Equation (1), when the pulse current is the same, the peak current density of the cathode increases with the decrease of the duty cycle. The excessive peak current density results in cathode over-potential and the Cu^2+^ ions near the cathode cannot be replenished in time. These results lead to severe concentration polarization and the formation of coarse grains. At the same time, if the cathode peak current is too high, the higher degree of side reactions, such as hydrogen evolution, generates H_2_ bubbles and forms holes because the H_2_ bubbles cannot be discharged in time, causing an uneven structure [23]. Therefore, we have observed a rough surface and coarse particles at *d_f_* = 20%. However, when the forward duty cycle of pulse current increases to 60%, the peak current density obviously decreases and significantly slows down the formation speed of Cu seed, resulting in inferior plating efficiency [19] and, in turn, a loose structure.

**Figure 5:**
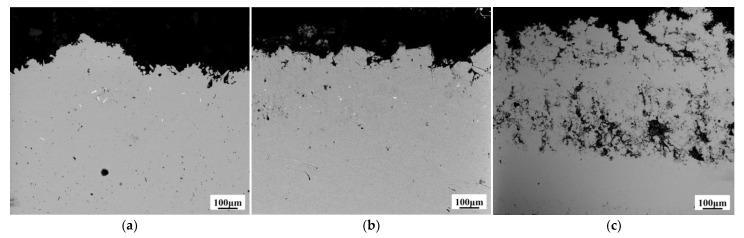
The cross-sectional SEM images of W–Cu composite coatings under different forward duty cycles: (**a**) *d_f_* = 20%, (**b**) *d_f_* = 40%, and (**c**) *d_f_* = 60%, (*J* = 2 A/dm^2^, *f* = 1500 Hz and *d_r_* = 5%).

**Figure 6:**
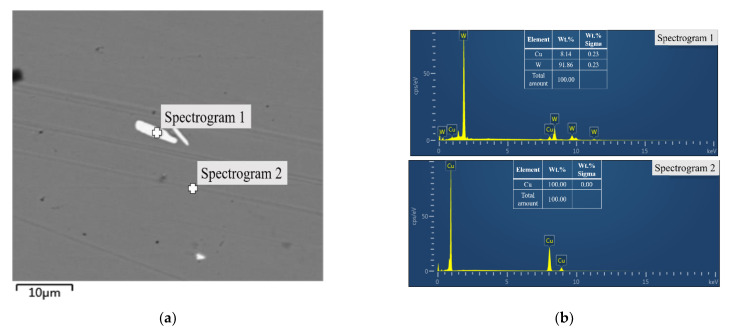
The cross-sectional SEM image (**a**) and EDS analysis (**b**) of W–Cu composite (*J* = 2 A/dm^2^, *f* = 1500 Hz, *d_r_* = 5% and *d_f_* = 20%).

In order to observe the internal structure of W–Cu composite coatings, composite coatings were cut perpendicular to the surface. Then the cross section was polished with sandpaper to test the cross-sectional morphology of samples. samples were also prepared using an ion thinner for TEM observation. Figure 5 presents the cross-sectional SEM images of W–Cu composite coatings with different forward duty cycles. All observed in the picture are composite coatings obtained by electroplating, and there is no substrate. The thickness of the composite coating is about 700 μm. It can be readily observed that the forward duty cycle of pulse current renders a significant influence on the microstructure of W–Cu composites. At *d_f_* = 40%, the composite coating is dense and uniform than the other two conditions. A loose structure is obtained at *d_f_* = 60%, which is consistent with Figure 4c. The EDS analysis of W–Cu composite (*d_f_* = 20%) shows that the uniformly dispersed white particles belong to the W dispersed in the Cu matrix (Figure 6). W particles distributed in the Cu matrix can be observed in Figure 5a,b, and the distribution of W particles in Figure 5b is relatively uniform and large in number. It can be also observed that there are more W particles dispersed on the surface of the composite in Figure 4b indicating that the W–Cu composite prepared at *d_f_* = 40% has a higher W content. However, W particles were not observed on the surface and cross section of the composite prepared at *d_f_* = 60% (Figure 4c and Figure 5c). This is due to the loose structure of the composite. W particles fall off from the Cu surface during the cleaning and drying process. Hence, the *d_f_* of 40% was selected for subsequent experiments because it renders a compact structure.

### 3.2. Effect of Reverse Duty Cycle on the Structure of W–Cu Composite Coatings

**Figure 7:**
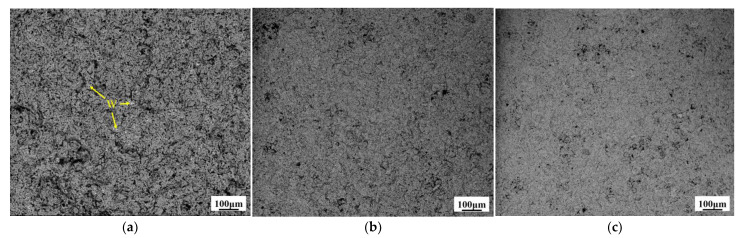
Surface SEM images of W–Cu composite coatings under different reverse duty cycles: (**a**) *d_r_* = 5%, (**b**) *d_r_* = 10%, and (**c**) *d_r_* = 15%, (*J* = 2 A/dm^2^, *f* = 1500 Hz and *d_f_* = 40%).

Figure 7 presents the effect of pulse current reverse duty cycle on the surface morphology of W–Cu composite coatings. The surface of the W–Cu composite became smooth and the particles were refined with the increase of the reverse duty cycle. The results show that the reverse pulse current is beneficial to the anodization dissolution of the cathode and caused the Cu grains smaller [19]. When reverse pulse current is applied, the coarse-grained Cu obtained by forward-pulse current plating loses electrons, turns into Cu^2+^ ions, and dissolves into the bath again. The concentration of Cu^2+^ ions is rapidly increased on the cathode surface, which is conducive to the use of high pulse current density in the subsequent pulse cycle to obtain a compact coating with fine particles.

**Figure 8:**
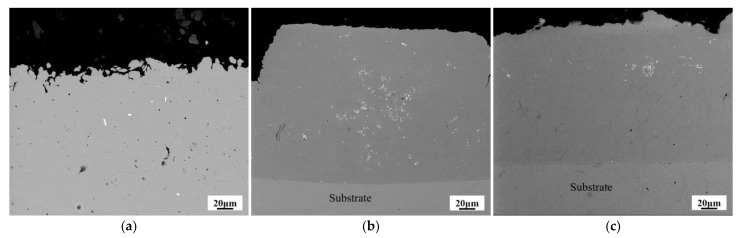
The cross-sectional SEM images of W–Cu composite coatings under different reverse duty cycles: (**a**) *d_r_* = 5%, (**b**) *d_r_* = 10%, and (**c**) *d_r_* = 15%, (*J* = 2 A/dm^2^, *f* = 1500 Hz and *d_f_* = 40%).

**Figure 9:**
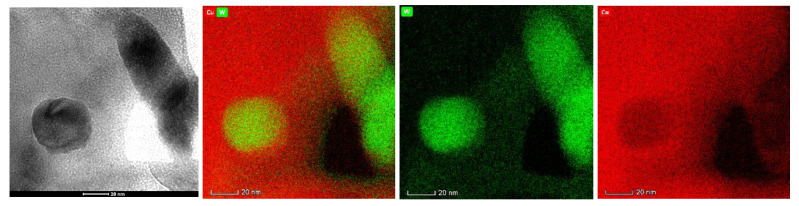
TEM image and EDS maps of W–Cu composite (*J* = 2 A/dm^2^, *f* = 1500 Hz, *d_f_* = 40% and *d_r_* = 10%).

Figure 8 presents the cross-sectional SEM images of W–Cu composite coatings with different reverse duty cycles. From the analysis in Figure 5, it can be derived that when *d_r_* = 5%, the thickness of the composite coating is about 700 μm. However, it can be estimated from Figure 8b,c that the thickness of the composite coating under different reverse duty cycles (*d_r_* = 10% and *d_r_* = 15%) is 190 and 170 μm. In order to observe and compare at the same multiple, the composite coating that is shown in Figure 8a has no substrate. The thickness of the composite coating decreases with the increase of the reverse duty cycle. This is due to the increase of the reverse duty cycle and the increase of the reverse electroplating time, which leads to an increase in the dissolution of the composite coating and ultimately to a decrease in the thickness of the composite coating. At *d_r_* = 5% (Figure 8a), the internal structure of W–Cu composite contains a large number of holes and the W particles are embedded in the coating. However, the distribution of W particles is not uniform, showing severe agglomeration. At *d_r_* = 10% (Figure 8b), W–Cu composites are dense and the concentration of uniformly distributed W particles is significantly increased. At *d_r_* = 15% (Figure 8c), the microstructure of W–Cu composite is more refine and uniform; however, the relative concentration of W particles is compromised. These results could be ascribed to the anodized peeling by reverse pulse current, desorbing the already adsorbed W particles from the coating. Figure 9 shows the TEM and EDS maps of W–Cu composite with *d_r_* = 10%, confirming that W nanoparticles are successfully embedded in the Cu matrix.

**Figure 10:**
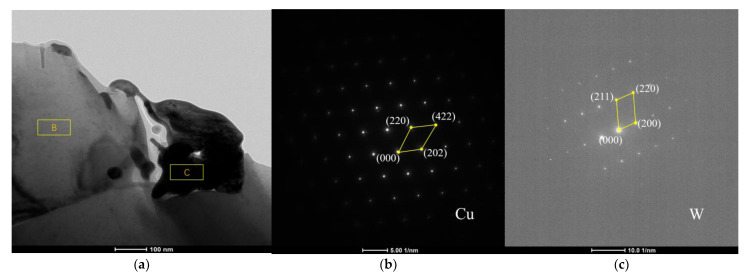
TEM images of W–Cu composite (*J* = 2 A/dm^2^, *f* = 1500 Hz, *d_f_* = 40% and *d_r_* = 10%): (**a**) TEM image of W−Cu composite; (**b**) electron diffraction pattern of position B in Figure 10a; and (**c**) electron diffraction pattern of Position C in Figure 10a.

Figure 10a is the TEM image of W–Cu Composite under a low-power microscope. It can be observed that W particles exist between Cu grains, and Cu grains are closely bonded. However, there are voids at the boundary between W and Cu, which will affect the properties of W–Cu composites. Figure 10b shows the electron diffraction pattern of area B in Figure 10a, and Figure 10c shows the electron diffraction pattern of area C, which further proves the existence and distribution of W and Cu phases in the composite.

**Figure 11:**
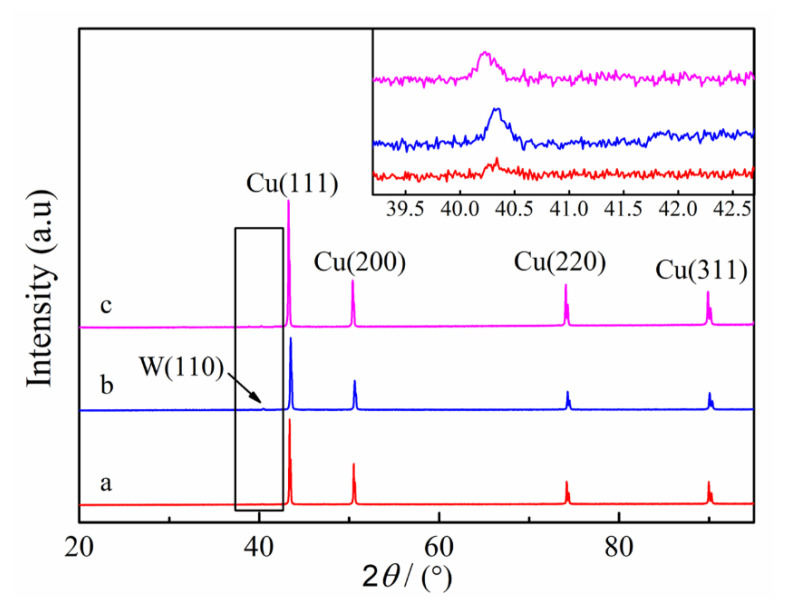
XRD patterns of W–Cu composite under different reverse duty cycles: (**a**) *d_r_* = 5%, (**b**) *d_r_* = 10%, and (**c**) *d_r_* = 15%, (*J* = 2 A/dm^2^, *f* = 1500 Hz and *d_f_* = 40%).

Figure 11 shows the XRD patterns of W–Cu composites with different reverse duty cycles. It can be seen that the diffraction peaks are consistent with the main peaks of Cu, corresponding to (111), (200), (220) and (311) planes of Cu. A small peak is observed at 2*θ* ≈ 40°, which corresponds to (110) planes of W. Apart from that, the other diffraction peaks of W were not obvious in XRD patterns. The inset in Figure 11 shows that the peak intensity of W (110) planes in the W–Cu composite (*d_r_* = 10%) is higher than the other two conditions, corresponding to relatively higher W content. The results of ICP show that the W content in W–Cu composites prepared under the conditions of *d_r_* = 5%, *d_r_* = 10% and *d_r_* = 15% is 1.16 wt.%, 8.33 wt.% and 2.97 wt.%, respectively.

### 3.3. Densification Mechanism of Pulse Electroplated W–Cu Composites

**Figure 12:**
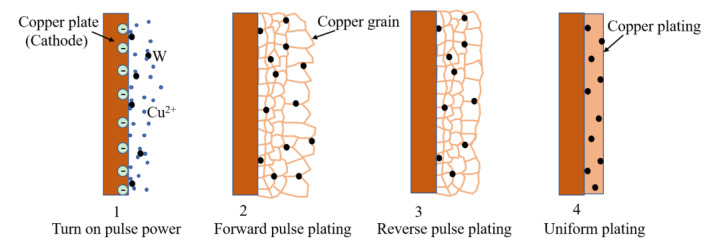
The densification mechanism of W–Cu composite coatings prepared by pulse electroplating.

The aforementioned results confirm that highly dense W–Cu composite coatings can be prepared by adjusting the forward and reverse duty cycles of the pulse current. The whole electroplating process is schematically illustrated in Figure 12.

The complete densification of W–Cu composite coatings is achieved in four steps. First, when the pulse power is turned ON, Cu^2+^ ions move to the cathode, and W nanoparticles are driven to the cathode due to the adsorption of Cu^2+^ ions. Due to the large specific surface area and surface energy of W nanoparticles, Cu^2+^ ions preferentially reduce on the surface of W particles to form Cu nuclei [17]. Second, the Cu nuclei continuously grow to form a Cu coating during the forward pulse. However, with the continuous growth of Cu nuclei, coarse particles are easy to form and Cu^2+^ near the cathode cannot be replenished in time, resulting in concentration polarization and formatting a rough and uneven surface [18,22]. Third, when the reverse pulse current is switched on, the cathodic Cu coating is dissolved due to anodization and Cu^2+^ ions are again dissolved in the plating bath [15]. At the same time, the desorption of W particles results in refined and uniform Cu grain size. Fourth, a uniform and dense W–Cu composite coating is formed by alternating the forward and reverse pulse electroplating at high frequency.

### 3.4. Microhardness and Electrical Conductivity of W–Cu Composites

The Vickers hardness and conductivity of W–Cu composites, prepared under *J* = 2 A/dm^2^, *f* = 1500 Hz, *d_f_* = 40% and different reverse duty cycles *d_r_* = 5%, *d_r_* = 10% and *d_r_* = 15%, are shown in Table 2. The polished W–Cu composite coatings were placed on the hardness tester, and the hardness of the samples was tested by using a pressure load of 200 g and a holding pressure of 15 s. The hardness values of five different positions were measured, and the average value was taken as the hardness of the W–Cu composite. The hardness values under different reverse duty cycles, i.e., *d*_r_ = 5%, *d_r_* = 10% and *d_r_* = 15%, are found to be 90, 127 and 103 HV, respectively, which are higher than the hardness of the experimental Cu cathode (85 HV). Hence, the hardness is improved by the incorporation of W particles (300–650 HV) and increased with increasing W content. At *d_r_* = 5%, the hardness of W–Cu composites is the lowest because of the porous structure. On the other hand, the electrical conductivity increased with the increase of the reverse duty cycle. The conductivity values of the composites prepared under different reverse duty cycles, i.e., *d_r_* = 5%, *d_r_* = 10%, and *d_r_* = 15%, are found to be 48.0, 53.7 and 56.5 MS/m, respectively. As expected, the conductivity of W–Cu composites is found to be lower than pure Cu (57.1 MS/m) due to the presence of W (17.7 MS/m) and the existence of holes. The structural analysis of W–Cu composite revealed that the microstructure of W–Cu composite is more uniform and compact at *d_r_* = 15%, forming a continuous Cu structure and facilitating the electron transfer process. Therefore, the conductivity of the W–Cu composite is highest at *d_r_* = 15%; however, the hardness decreased to 103 HV. Hence, *J* = 2 A/dm^2^, *f* = 1500 Hz, *d_f_* = 40% and *d_r_* = 10% are selected as optimal processing parameters for pulse electroplating.

## 4. Conclusions

By adjusting the forward and reverse duty cycles of the pulse current, W–Cu composite coatings with uniform microstructure can be directly prepared by a one-step electroplating method using nano-W powder as a raw material. At *d_f_* = 40% and *d_r_* = 10%, W content in W–Cu composite was found to be 8.3 wt.%, which increased the hardness to 127 HV without compromising the electrical conductivity (53.7 MS/m). Although the higher electrical conductivity of W–Cu composites can be maintained by decreasing the W content, the hardness cannot be maintained. Hence, the optimal pulse plating parameters are found to be *J* = 2 A/dm^2^, *f* = 1500 Hz, *d_f_* = 40%, and *d_r_* = 10%. The densification mechanism of the W–Cu composite coatings mainly relies on controlling the forward duty cycle, increasing the peak current density, switching on the reverse pulse current, and reducing the concentration polarization. Overall, the present study demonstrates that the electrical and mechanical properties, which mainly depend on the microstructure and phase composition, can be tuned by optimizing the pulse plating parameters. This lays a good foundation for the application of such electrical contact materials widely used in electronics fields.

## Figures and Tables

**Table 1:** The parameters of the electroplating process.

Conditions	Parameters
Current density (*J*)	2 A/dm^2^
Frequency (*f*)	1500 Hz
Forward pulse duty cycle (*d_f_*)	20–60%
Reverse pulse duty cycle (*d_r_*)	5–15%
Temperature	30 ℃
Plating time	2 h

**Table 2:** The comparison of Vickers hardness and electrical conductivity of W–Cu composites under different reverse duty cycles: *d_r_* = 5%, *d_r_* = 10%, and *d_r_* = 15%.

Properties	Cu	W	W–Cu Composite (*J* = 2 A/dm^2^, *f* = 1500 Hz, *d_f_* = 40%)		
			*d_r_* = 5%	*d_r_* = 10%	*d_r_* = 15%
Vickers hardness (HV)	85	300–650 ^1^	90 (SD ^2^ = 1.75)	127 (SD = 0.84)	103 (SD = 1.21)
Electrical conductivity (MS/m)	57.1	17.7	48.0 (SD = 2.05)	53.7 (SD = 0.75)	56.5 (SD = 0.85)

^1^ The hardness of tungsten is different due to the different crystal state and crystallization mode of tungsten. ^2^ SD is the standard deviation of the value of five measurements.

## Data Availability

Data available in a publicly accessible repository.
